# Third sector perspectives on community link worker referrals in social prescribing: a realist analysis

**DOI:** 10.3389/fpubh.2025.1645353

**Published:** 2025-10-08

**Authors:** Helen Allbutt, Donald Maciver, Alison Leitch, Anne Crandles, Linda Irvine Fitzpatrick

**Affiliations:** ^1^School of Health Sciences, Queen Margaret University, Edinburgh, United Kingdom; ^2^Edinburgh Health & Social Care Partnership, Waverley Court, Edinburgh, United Kingdom; ^3^Edinburgh Napier University, Edinburgh, United Kingdom

**Keywords:** social prescribing, community link workers, third sector, community organisations, realist study

## Abstract

**Introduction:**

Community link workers (CLWs), also known as social prescribing link workers, connect individuals to community support, much of which is provided by third-sector organisations (TSOs). TSOs are common referral destinations for CLWs, yet the relationships between CLWs and TSOs remain under-explored.

**Method:**

This realist study investigated TSOs’ perceptions of link working, focusing on referral and collaboration dynamics. Conducted across 22 TSOs in Scotland, it involved in-depth interviews with TSO staff and consultations with CLWs and managers, analysing data via realist heuristics to identify contexts, mechanisms, and outcomes.

**Results:**

Targeted referrals by experienced CLWs, with follow-up, and strong TSO-CLW relationships, improved client health, wellbeing, and independence while reducing reliance on statutory services. These processes fostered professionals’ trust, satisfaction, and innovation, creating a positive feedback loop. Conversely, inconsistent referrals, such as over-referral or scattergun approaches, compounded by resource constraints and perceptions of inequitable partnerships, led to suboptimal client outcomes, dissatisfaction, and weakened collaboration. Important contexts included extreme resource pressures, varying CLW expertise, and perceptions of power imbalances.

**Discussion:**

Effective social prescribing needs sustained TSO-CLW collaboration, supported by third-sector investment and tools for equitable partnerships. Rather than a referral process, social prescribing should be envisioned as a community of practice, defined by relationships, a common purpose and shared responsibility for challenges and solutions. Future models should prioritise strengthening statutory and third-sector trust and collaboration.

## Introduction

Social prescribing describes a variety of approaches by which individuals are linked to resources and services within communities to improve wellbeing (1). In Scotland, referrals generally, but not exclusively, come from health care professionals working within primary care settings, predominantly general practitioners (GPs). Social prescribing has strong roots in practice in the United Kingdom (UK) that trace back to community-centred ways of working to promote health and wellbeing (2). Different models of social prescribing operate even within and across the four nations of the UK with access in Wales, for example, via local authorities, community resources, healthcare and self-referral (3). Whilst England was first to integrate social prescribing into national policy, other countries throughout the world are adopting similar approaches at pace and scale (4).

The “prescribing” of community activities is regarded as a means of encouraging person-centred care, but also to deal with the large volume of repeat appointments for “socially determined” health concerns (5). In 2021, for example, between 25 and 50% of GP appointments in Scotland were estimated to focus on “non-medical” issues such as social isolation, deprivation, or financial struggles (6).

Social prescribing is based on the concept that health inequalities lie mainly outside of healthcare and result from the socio-economic conditions in which people are born, grow, live and work, otherwise known as the social determinants of health (7). Socioeconomic factors (such as educational attainment, occupation and income) have a well-established dose–response relationship in which health outcomes increase along with greater social advantage and vice versa (8). People living in socioeconomically deprived areas are at higher risk of developing multiple health conditions and to have co-occurring conditions at a younger age (9).

Deprived communities have also borne the brunt of the wider impacts of the COVID-19 pandemic including financial hardship, job losses, food poverty and social isolation (10). Social prescribing is viewed as a mechanism to address unmet need which is likely to be higher and more complex among those living in socio-economically deprived communities (9).

Various models of social prescribing exist (11–13) but most comprise the use of a “community link worker” (CLW). CLWs are generalist practitioners, typically attached to GP practices, who support people to identify what matters to them, helping them access and use non-medical sources of support in local areas (14). Such sources may include support for mental health, benefits, welfare and legal advice as well as opportunities for the arts, gardening, outdoor activity, learning, music, exercise, etc. Many of these sources of support are provided by third sector organisations (TSOs) representing a range of non-statutory, non-profit, community-based organisations, including charities, social enterprises, and voluntary groups. While terminology and structures vary across countries, the third sector, however defined, serves as a key partner in social prescribing and one of the most common destinations for community link worker referrals (15).

### Evidence on social prescribing

Previous studies have examined the impact of social prescribing across the UK and internationally. Many focus specifically on the community link worker model (16–18). Investigations comprise systematic reviews (19, 20), realist evaluations (21, 22), and case studies of CLW programmes (12, 23). They report promising findings such as reduced pressure on primary care, alleviating social isolation (24), improved individual self-esteem, confidence, mental wellbeing, quality of life and reduced symptoms of anxiety and depression (11, 12, 19, 20) as well as inconsistent results including use of less robust and non-standardised measures (25), and small scale studies (26). Economic evaluations of social prescribing are also emerging (27). Overall, they find that social prescribing can cut secondary care costs and reduce pressure on health service use.

Realist evaluation offers a way to understanding how interventions function in the real world, and how they can be improved and adapted (28). Several realist studies have been conducted on social prescribing and the impact of CLWs. Husk et al. (29), for example, considered the evidence of existing social prescribing interventions and aimed to clarify the principles underpinning social prescribing practice. Using a realist lens, Tierney et al. (30) reviewed CLW models to determine how the CLW role might be implemented optimally in practice. Bertotti et al. (21) evaluated a social prescribing pilot in a London district to unpack the contextual factors and mechanisms that operated at different stages of the social prescribing “intervention.”

Whilst social prescribing has been widely studied, partnership working between CLWs and third sector organisations (TSOs) remains poorly understood (6, 31, 32). TSOs are essential to the success of social prescribing, as they provide many of the services and community-based resources that CLWs rely on to support individuals. Yet little is known about how these partnerships function in practice. Understanding this dynamic is important for improving the effectiveness and sustainability of social prescribing models. This study addresses this gap by examining CLW-TSO partnerships, with a particular focus on the perspectives of TSOs as the recipients of referrals and the organisations responsible for delivering services following referral.

### Taking a realist approach

The heterogeneous nature of CLW programmes makes evaluation challenging. Realist approaches to programme evaluation recognise the importance of context in shaping outcomes and seek to consider the circumstances or conditions in which an intervention operates (28). For this reason, they are particularly well suited to evaluating complex programmes (28, 33).

Pawson and Tilley (28) who first developed the realist approach, argued that in order to improve the effectiveness of programmes, research needs to identify what works, for whom, under what circumstances, and how. By clearly articulating the “programme theory” behind an intervention, policymakers can gain a deeper understanding of the factors driving differences in implementation or outcomes, enabling them to adjust and improve programmes as needed.

Realist evaluation aims to explain the complex operations of interventions: the context within which a programme is implemented, the underlying mechanism(s) that generate outcomes, and the outcomes that are sensitive to variations in both mechanisms and contexts (34). Constructing C-M-O configurations is a heuristic tool used in the method to unpack the intrinsic theory of the intervention, otherwise known as a “programme theory.” The evaluation produces a detailed understanding of causation, i.e., this outcome (O) is generated due to this mechanism (M) which is triggered by, or best works in, this context (C) (33).

### CLW Edinburgh study

In 2017, the Scottish Government committed to introduce 250 CLWs to work in GP surgeries in Scotland’s most deprived communities (35). The Edinburgh CLW programme was an “early adopter” site with 25 CLWs working in primary care practices, employed by different community groups. A delivery model was set up whereby CLWs were expected to work with individuals for four to six sessions prior to referral to community organisations.

Local evaluation revealed positive outcomes (35), with the Edinburgh CLW service seen as a “demonstrator” site, receiving awards for its operations. Being relatively “young” in its implementation, the service was keen to improve and was open to more systematic investigation. Unlike previous research that reviewed CLW perspectives, this study examined the views of the CLW service from the standpoint of TSOs.

### Ethics

Ethical approval for the study was obtained from the Queen Margaret University Ethics Committee, Edinburgh (reference: 2024/15). All participants provided written informed consent.

## Method

A realist evaluation using qualitative methods was conducted. A study team was formed combining members with managerial oversight of the local CLW programme, experience in intersectoral partnerships, and researchers. As part of the realist approach, our first step involved developing initial programme theory(ies) on the referral process between TSOs and CLWs. To do this, we met managers of the CLW service to better understand the CLW service and elicit their reasoning about how the programme worked. We also reviewed published and grey literature on social prescribing but found limited research on referral processes to TSOs.

### Sample

The sample comprised 22 TSO staff. Participants were selected through purposive sampling, focusing on TSO sites where, according to CLW service managers, CLW referrals had been handled smoothly, as well as those where hurdles had been reported. Issues had seemingly arisen in only a small number of sites.

### Fieldwork and analysis

All interviews were conducted by the first author who had no preexisting relationship with participants. Interviews were audio-recorded, and detailed field notes were compiled. The interviews elicited insights into the conditions, mechanisms, and contexts under which CLW referrals to TSOs were more (or less) successful. Participants provided accounts that illuminated contextual nuances, outcomes following referral, and causal explanations. Analysis was conducted concurrently with data collection to test and refine emerging programme theories. To mitigate the risk of biasing responses, we employed open-ended questions about the referral process while also inviting participants to critique provisional explanatory assumptions (36). By the end of the data collection, few new insights were emerging indicating that the key patterns and mechanisms had been sufficiently explored.

Data were imported into NVivo 14. Material included transcripts, memos, notes, and minutes of meetings. Analysis followed realist techniques, Framework-style analysis and coding (37). The procedure involved familiarisation of data, identification of codes and categories, indexing the data according to the CMO structure, charting, and mapping and interpreting the data (37). In keeping with a realist philosophy, we interrogated the data for causal explanations of outcomes and the contextual factors at play which triggered mechanisms. This was an iterative process. The research team (authors) collaborated to review emerging CMO configurations, debate interpretations, and negotiate consensus on knowledge claims. Engagement with the CLW service was maintained to ensure the research recommendations were relevant and actionable by them. To this end, findings and recommendations were reviewed with a group of CLWs (*n* = 3) and a group of CLW managers (*n* = 4). The lead author, external to the CLW service and not embedded within its operations, retained autonomy in interpreting data and formulating conclusions.

## Results

### Participants

We interviewed 22 participants from 22 different organisations (see Table 1). The organisations included in the study varied in size, purpose, types of support and resources. Some provided “crisis-type” help such as debt advice, homelessness and housing support, whereas others sought to engage individuals in outdoor opportunities, arts and crafts, exercise, and community groups. Still more offered specialist services such as counselling, mental health and wellbeing support and trauma therapy. They represented a comprehensive selection of TSOs in the local area to ensure a range of TSOs were included.

**Table 1:** Participant demographics.

Participant	Organisation/role	Gender	Age group
1	TSO	Female	56+
2	TSO—Crisis*	Female	46–55
3	TSO—MH	Female	26–35
4	TSO—MH	Female	56+
5	TSO	Female	36–45
6	TSO—Employer+	Male	56+
7	TSO	Male	26–35
8	TSO	Female	46–55
9	TSO—Employer+	Male	46–55
10	TSO	Female	46–55
11	TSO—Crisis*	Female	26–35
12	TSO	Female	56+
13	TSO—Crisis*	Female	26–35
14	TSO—Employer+	Female	46–55
15	TSO—Crisis*	Female	46–55
16	TSO—Employer+	Female	36–45
17	TSO	Male	26–25
18	TSO—Employer+	Male	46–55
19	TSO—Employer+	Female	46–55
20	TSO—Crisis* + Employer+	Male	56+
21	TSO—Employer+	Female	26–35
22	TSO—Employer+	Female	56+

*Crisis refers to TSOs that offered support for immediate needs, for example, debt relief, food banks, housing advice, homelessness.

+Employer refers to TSOs that employed CLWs.

MH refers to TSOs providing mental health services.

### Findings

The narrative findings are organised into two sections: (1) the point of referral and the immediate interactions between the CLWs and TSOs; (2) following referral including client outcomes, perceptions of success for the CLW service and partnership working. The realist matrix and summary of CMO configurations are at the end of the findings section.

#### The point of referral

##### What is a successful referral?

TSO staff reported that most referrals from CLWs were suitable. Successful referrals from a TSO perspective were those where individuals were in the “right place” and ready for the services they received. Engagement in community activities was seen as the means by which clients were willing and able to take independent actions to manage their own health and care:

*We want to find the right people at the right time and help them in the right way. We want to support the people with the greatest potential for their life to significantly change. If we put a lot of work in, even if it takes 18 months or more, that person’s life is going to be much better, and they are going to not need that level of support ever again. It needs to take them to a much better place. P17*

CLWs were seen to offer valuable preparatory support, helping individuals move forward and begin considering how to improve their lives. TSO staff highlighted the importance of this early work in shifting people away from feeling stuck, so that by the time they engaged with their services, they had already begun to reflect on making changes to their lives. This was seen as essential groundwork, moving individuals beyond the stage of contemplation towards taking positive action:

*I suppose for us that’s about link workers starting the conversation with people, so folks themselves are getting really clear about what it is that they want to see different in their lives. Because actually that’s what makes a better referral is the end point, and then work backwards. That then determines which service we might actually support somebody into. P12*

##### Referral transactions

According to TSO staff, appropriate linking depended on CLWs’ empathetic understanding of client needs. From their perspective there were varying levels of link worker experience with greater experience improving referral quality including CLW decision-making about the types of services clients required, the timing of those services, and whether further involvement by CLWs was necessary. Targeted and customised placing of clients by link workers was preferred over a “scattergun” approach. TSOs favoured positive relational interactions with CLWs and identified these as important mechanisms in facilitating successful referrals:

*We have really close relationships with the link workers and some of that is, actually one of the link workers there, used to work here. So she knows intimately what we do. P13*

*I think that certain community link workers probably know us more and know where the groups are and they have been around for some years. They know what they are doing. So I would not say it’s widespread for all community link workers, I would say, it’s only certain ones that aren’t so good. P5*

TSO staff reported that whilst some clients needed simple navigation to one or more agencies (e.g., food banks, debt relief), many others required much more in-depth support in their transition to the third sector. When that support was provided, positive engagement of clients with community activities was considerably more likely. This “handholding” or “buddying” role was viewed positively and perceived as a key mechanism to support attendance and reduce drop out:

*(The community link worker) they’ll bring people along that are really quite isolated… they will come into the group with them, and just sort of make sure they feel comfortable. So that then they start attending. So I think that’s what the community link workers are really good at. So I think that just sort of handholding into the service can really help. P16*

*I know from experience where community link workers have been able to buddy along definitely made a difference. Sometimes, support workers have been able to do that, or for the people just bringing a friend, but it takes a hell of a lot of courage to come along to the unknown. Sometimes, we are getting, really quite recently, as well, people coming along for one session and not coming back. They’ve been able to build up to coming to one, and then sometimes if they miss a few, as well, feel like they cannot come back. P5*

However, TSO staff reported that some CLWs expected clients to take on that initial step. There was also a perception that latterly CLWs may be under too much pressure in their role to consistently accommodate a “handholding” task for every individual. When overwhelmed by work, CLWs appeared to reduce their level of interactions with TSOs:

*It used to be more common for them (CLWs) to maybe take people along to groups, whereas now due to the pressures, they just do not have the capacity to do that. So, it’s a rare circumstance that they’ll actually do more than just that initial drop-in. P9*

*This (CLW) is very much about, you know, you need to do this for yourself, because if you are, you know, it’s about your motivation. I do not think she does that (accompany clients) very often. P22*

##### Volume of referrals

Some TSOs reported over-referral and/or under-referral from the CLW service. When experiencing high rates of referral, TSOs made great effort to accommodate clients. Only occasionally did TSOs place restrictions on entry or set up waiting list systems. This was particularly true for crisis-type TSOs:

*We support about 300 to 400 people a year. Quite a big percentage of those referrals are actually coming through community link workers. We do have a waiting list. That waiting list at this moment is up to one month but we always do keep a little capacity…each practitioner is actually able to take maybe one more referral in case of an emergency. P13*

However, when individuals required mental health support, levels of need for these services far outweighed their availability. TSOs reported some CLWs tried to “game the system” for clients by referring them to several different services at the same time. Such “scattergun” practice was universally criticised as it served to increase waiting lists, duplicate staff time and cause client confusion:

*There is also a sense that people are being referred to multiple services at once, which can also be quite unhelpful because sometimes when we are talking to the person, they are a bit confused as to who’s contacting them because there’s been a few, or like, what service are you? Or sometimes because of the waiting list, we can be like buses, there’s no support and then suddenly all of the support is happening at once, which can then make certain support just either redundant or unhelpful because they are getting different types of information. So I think that’s where things get a bit tricky is not necessarily knowing what conversation the person has had and where else they have been signposted into as well*. *P3*

Under-referral, irregular referral patterns or lack of follow up communications eroded trust for TSOs. Some TSO staff said that CLWs relied too heavily on familiar TSO provision to expedite client referral rather than exploring the full range of options available locally. These were important mechanisms for less favourable outcomes, and a source of frustration for TSOs:

*I would say we are quite remote at the moment and disengaged from them (CLWs). We’re quite happy to work in cooperation but I would say it’s quite few and far between in terms of referrals that we get. And then it’s random about where they might refer, you know? Well, I’m not saying all, but some link workers have got to follow up, but very few do really, you know. They are so intent in getting the referral out of their inboxes that’s where they are under pressure, you know, obviously to do that. P1*

*In terms of referrals, it definitely goes in waves. It feels like it might be that sometimes we are forgotten. Having said that, the success rate for link worker referrals is very low. I think there’s either multiple attempts from myself to follow up and initiate contact and make appointments, which does not follow through, or there’s an initial interest and then it just disappears. P7*

##### Increased needs since COVID-19

The heterogeneity of TSO provision helped to meet the multiplicity of individual needs which, according to participants, were greater and more complex since COVID-19. Higher levels of need were also associated with cuts in funding and a retrenchment in statutory services. These were important contextual factors:

*I’ve worked in health and social care and the council and there’s huge cuts…So yeah, it just means everybody’s trying desperately to look after needs*—*too many needs, which means that people get missed all the time. There’s just*—*there’s not enough services and too many people. P4*

*I think what we are seeing is people who have more complex difficulties and that is as a result of folks not probably being able to access statutory services. So more complexities, mild to moderate mental health issues becomes moderate and that kind of thing. P12*

#### Following referral

##### Client outcomes

For TSOs, client engagement in services and increased self-confidence led to longer-term benefits in terms of individual health and wellbeing. Several participants reflected that the collaborative work between CLWs and TSOs in matching clients to the most appropriate placement led to wider outcomes such as reducing pressure on GP services:

*(Client) did not speak to anybody when he first came for about 6 months, but he just wanted to be around people. He was on loads of medication. And he was using the doctor, he was always going to his doctor. So now he has somebody to talk to here. And now he does not need to see his GP. He does not need half his medicine now… P8*

*You know, she’s (client) is the first one to admit she was one of these people who was regularly making appointments at the GP practice because you know, she did not really realise, you know, other ways that she could improve her health and well-being. P21*

While the CLW service was seen primarily as a short-term intervention, TSOs provided several examples where extended partnership working with CLWs had helped to achieve client outcomes. Whilst these could be challenging to deliver, there was also job satisfaction in being able to make a difference:

*I saw this client for quite a long time …she could not access any benefits. So, one of the link workers referred her in, and she speaks quite limited English, so we needed interpretation…Basically we worked together and because her immigration status changed, she had access to public funds. So, it was a lot more collaborative and also, she asked us, basically, to update the link worker and also to be in touch with her social worker. So, I was like, back and forth emailing the social worker, because I think the social worker was asking her what was happening, even though we had interpretation. So, it’s just*—*yeah, I think just the different organisations working together worked really well here. P11*

These instances highlighted the value of multi-agency working but also revealed that meaningful progress often depended on sustained support beyond what CLWs could offer.

##### Perceptions of CLW service success

Some TSOs explicitly distinguished between what they perceived as *outputs* delivered by the CLW service and *outcomes* they generated through adopting an asset-based community development (ABCD) approach. This distinction highlighted perceived differences in organisational values and practice models, with TSO staff critiquing what they saw as a referral-driven culture throughout the statutory sector, and particularly within the CLW service:

*I think there’s something about the link worker network really being focused on referrals, which is almost irrelevant, frankly. It reflects nothing. We deliberately try to take a community development approach, a mindset informed by ABCD principles for instance although we sometimes have to operate with funding that traditionally has been used for quite paternalistic services, or advice services…Social prescribing should be moving beyond linking and moving beyond prescribing, moving towards a more sustainable, bigger change. P17*

Concerns were raised about the narrowness of reporting frameworks for the CLW service, which emphasised service engagement as a measure of success without necessarily capturing whether meaningful change had occurred:

*(The CLW service) are very proud of what they have achieved, but all the outcomes towards the end of the (CLW service) report were that link offered, engaged. So, that’s their top thing, that people engage with the service, which does not mean anything necessarily happened, but they engaged with the (CLW) service… I think that’s a battle or a change of attitude that hopefully those link workers would contribute to. P6*

This reflected a further tension between statutory services’ focus on measurable, process-driven outputs and TSOs’ emphasis on holistic, relational outcomes.

##### TSO contributions to client health and wellbeing

TSO staff expressed frustration that their contributions to public health and wellbeing were not always fully recognised, despite offering approaches that they argued were completely orientated towards supporting people with complex and long-term needs. Participants described the importance of supporting individuals to develop their own goals, coping strategies, and sense of resilience; work which, although preventative and effective, was perceived as somewhat at odds with a traditional medical model:

*What can we do that’s either in one-to-one support or group support for people, because we know group support can actually be great for people with chronic pain, for instance. How do we get them into a conversation about pain management, nutrition, wellness, well-being, mindfulness, so they can better understand themselves. I know that sounds very un-medical all of a sudden, but I do not understand why the medical system does not spend longer saying that there’s a whole job for people to understand themselves and their goals and how they see themselves in life because that’s the sort of resilience people need. P17*

##### Perceptions of partnership working

Some TSO participants felt that third sector organisations continued to be treated as peripheral, rather than equal, partners within health and social care systems. This was reflected in both structural inequalities (such as lower funding and insecure commissioning) and cultural assumptions about the value and legitimacy of community-based support:

*Because we can offer things that you do not necessarily get from statutory services…for me this is around the how do you get this kind of work embedded within the wider health and social care partnerships, as opposed to a kind of nice to have. So I think that’s an ongoing issue. I think there is increasing awareness of this kind of work and the value of it. But I think it’s an ongoing battle. P12*

Several participants spoke candidly about power dynamics within collaborative partnerships, perceiving statutory bodies as controlling and at times patronising. There was frustration that third sector organisations were often expected to deliver services more cheaply, with better outcomes, while receiving less recognition or influence in decision-making:

*The statutory body’s idea of a partnership is, as in a slightly patronising way, as doing a wee bit of us doing a wee bit of work for them and you know, they probably tick a box thinking that’s that when the reality is we do it for less money than they do it and generally we do it better. P18*

These reflections suggest that while social prescribing initiatives have created opportunities for closer collaboration between statutory and third sector services, significant cultural and structural tensions remain. Differences in organisational ethos, conceptions of care, and definitions of success continue to shape the experiences and positioning of third sector providers within integrated care systems.

##### Governance issues and funding

TSOs reported strong connections between CLWs and primary care with CLWs operating as an effective bridge. CLWs were governed in this study by a “matrix model” whereby they were attached to GP practices, managed by a network of senior staff but employed by different TSOs. The latter helped to embed link workers securely in local communities. Balancing consistency and flexibility within such a heterogeneous structure, however, was problematic. Some TSOs challenged whether link workers were constrained by such arrangements: keeping client engagement to a limited number of contacts, juggling the demands of different managers, and lacking scope to pursue collaborative and/or innovative initiatives:

*It’s matrix management and it’s complicated and there are always tensions in that. It can be fraught because the link worker ends up with three bosses in effect. P9*

*We did one project and partnership with a link worker. She applied through the NHS fund, which was brilliant, because it took a bit of pressure off of us. So having her there, there was a really good turnout. So that was around the time we felt that we are seeing a lot more people in crisis, so we were looking at that gap that was existing. So the hope was that it would develop but it just wasn’t possible. She had to stop. P5*

We asked TSOs about information governance challenges, including problems with data sharing, but few reported issues. In fact, all TSOs appeared knowledgeable about data sharing protocols and ensuring confidentiality of client information.

A major contextual barrier for TSOs was funding. It was short-term, inflexible, with few revenue streams, particularly to cover core costs. Alarm was expressed about sustaining services when widespread cuts via the Integrated Joint Board were announced. This critical contextual factor permeated some discussions. Not only were TSOs in danger of closing altogether but the reduction in community support would also severely impact the CLW service:

*The whole sector has collapsed quite a lot since the IJB funding decision. The link workers will have less places to refer to and there’ll be the same amount of workers dealing with, I do not know, how many times the volume of people being referred. So the knock on effect is going to be, I mean it’s going to catastrophic if they do not do anything about it and they actually do allow these 64 charities just to fold, that’s going to be look really really shaky and the NHS are going to be overwhelmed. Social work are going to be overwhelmed. The prisons are going to be, you know, and all of these services are already pushed to their limits. P10*

This fragility of funding was further reflected in the power imbalance between TSOs and statutory services. Some TSOs felt that whilst the CLW service had created more collaboration increasing trust between sectors, the effect of cutting core funds exposed the lack of real partnership working. These perceptions represent challenges for achieving collaborative outcomes in the long term:

*There’s no acknowledgement certainly at the deputations some of the ignorant and arrogant statements that were made by Council and NHS officers were to be believed in terms of the lack of acknowledgement of the third sector in its role. It’s a completely inequitable situation and it is about forcing people who are in an unequal situation just now into a crisis situation where they will make higher demands on the services. So if they think they have got it bad with waiting lists and demand on the services now, just wait till they stop the 64 organisations. P2*

#### Summary of findings and realist matrix

Table 2 comprises the realist matrix, displaying the contextual factors, mechanisms, and short- and long-term outcomes.

**Table 2:** Final realist matrix.

Contextual factors	Mechanisms	Short-term outcomes	Long-term outcomes
Post-COVID challenges impacting communities and services High levels of individual need CLWs with varying levels of experience and knowledge of available community provision CLWs working under significant pressure in their roles CLW service monitoring practices focused on referral numbers, which may contrast with the asset-based community development (ABCD) approach of TSOs Matrix management structures for CLWs, contributing to role complexity Short-term and fragile funding arrangements for TSOs Differing organisational cultures and power imbalances between statutory services and the third sector CLWs linked to local GP practices in areas of socio-economic deprivation A supportive policy environment promoting social prescribing, enablement, empowerment, and recovery approaches	*Favourable*		
	CLWs acting as empathic navigators moving clients towards most appropriate community supports CLWs providing handholding support (accompanying clients to groups) CLWs developing positive relationships with TSOs CLWs considering full range of local provision, not just favoured TSOs CLWs with enough expertise and community knowledge to match clients to appropriate placements Longer-term CLW client contact (when appropriate) TSOs as CLW employers: embedded within local community Stable TSO funding	Needs-based client placements Clients mentally in “right place” for services Increased client motivation to take positive action Reduced drop out and non-attendance Sustained client engagement in TSOs Increased client confidence in CLW and TSO services More facilitative environment for TSO/CLW collaboration	Client receives right supports Increased client health and wellbeing Reduced GP and other statutory support attendance (e.g., police, emergency, social work) CLW and TSO job satisfaction Increased TSO trust in CLW service Increased innovation, collaboration and partnership working With stable funding, the third sector can offer a rich range of placements for clients
	*Unfavourable*		
	Over-referral by CLWs Under-referral by CLWs Failure by CLWs to follow up to TSOs after initial contact Scattergun and multiple referral practices Differing understandings of what constitutes CLW service “success” TSO perceptions of inequitable partnerships and unequal power compared to statutory services CLWs having too many managers Cuts to TSO funding from the Integrated Joint Board	Less effective client placements Increased waiting lists across services Particularly long waiting list for mental health services TSOs overloaded CLW reliance on preferred TSOs may limit other beneficial solutions for clients Duplication of staff time due to scattergun approach Client confusion if too many services or too long waiting lists Absence of a unified team ethos or sense of collective purpose between TSO and CLW services Less facilitative environment for TSO/CLW collaboration	Client dissatisfaction Clients do not receive right support with adverse impacts on health and wellbeing Increased use of GP and other statutory support attendance (e.g., police, emergency, social work) Poorer CLW-TSO relationships Decreased TSO trust in CLW service Reduced innovation, collaboration and partnership working When resources are cut there are fewer community options for clients to attend

Context: pre-existing individual, interpersonal, institutional or infrastructural factors of the CLW programme; mechanism: causal forces, processes, or interactions that generate change within the CLW programme; outcomes: short- or long-term impacts resulting from the interaction between mechanisms and contexts of the CLW programme.

The analysis found that collaborative and well-informed referral practices, underpinned by targeted referrals, appropriate follow-up, and high CLW expertise, alongside strong and equitable CLW-TSO relationships and stable resources, ensured effective client placements and sustained client engagement with TSO offerings. This led to enhanced client health, wellbeing, and independence, and reduced client reliance on statutory services. When these processes were working well, they fostered trust, professional satisfaction, and innovation between TSO and CLW services. This created a virtuous circle, providing the milieu in which the entire system could thrive.

Inconsistent referral practices (encompassing over-/under-referral, scattergun approaches, and lack of follow-up), disrupted by resource constraints and perceptions of inequitable partnerships and perceived inconsistency in organisational values, weakened CLW-TSO collaboration and hindered effective client placements. Less successful referrals led to client dissatisfaction and continuing reliance on statutory services. These practices resulted in misplacement of clients and missed opportunities for client engagement with the right TSO. Weakened CLW-TSO relationships further reduced trust, collaboration, and innovation.

Interacting layers of context were present, including elevated community needs, resource constraints (particularly for TSOs), varying CLW expertise, government priorities, contrasting organisational priorities, differing values, historical and longstanding power imbalances between statutory and third-sector services, and operational pressures. These contexts influenced effective link working practice, collaboration and TSO service delivery.

## Discussion

This study focussed on the relationships between CLWs and TSOs revealing what works, for whom, and under what circumstances. Drawing primarily from the perspectives of TSO staff, this study uncovered important contexts, mechanisms, and outcomes. With few CLW studies reporting the views of TSOs, this work provides insights into an area of importance for understanding social prescribing and the CLW model.

TSOs identified successful referrals as those where experienced CLWs had practised targeted placement with appropriate follow up alongside strong CLW-TSO relationships and stable resources. These factors led to engagement of clients with TSO services and over time to improvements in clients’ health, wellbeing, and independence, while reducing reliance on statutory services. Successful processes fostered TSO-CLW trust, professional satisfaction, and innovation, creating a positive feedback loop. In contrast, inconsistent CLW referral practices, such as over-/under-referral, scattergun approaches, and lack of follow-up, when compounded by resource constraints and perceptions of inequitable partnerships, weakened CLW-TSO collaboration. These practices led to suboptimal client placements, client dissatisfaction, and continued client dependence on statutory services. Wider contextual factors were also identified, including resource pressures, varying CLW expertise, and longstanding power imbalances between statutory and third-sector services, affecting collaboration and service delivery.

The core theory developed from this study is that long-term, mutually beneficial engagement between CLWs and TSOs contributes to more appropriate, timely referrals, better client matching to services and enhanced client outcomes. These outcomes include reduced GP visits, better client engagement with TSO services, improved physical and mental health and increased client self-management, all of which are central to the aims of social prescribing. When CLW-TSO processes are functioning effectively, they cultivate trust, professional satisfaction, and innovation between TSO and CLW services. This, in turn, creates a positive feedback loop that supports the overall success of the system.

A key implication arising from this analysis is that social prescribing initiatives cannot be operationalised as a linear referral mechanism, i.e., a process of moving individuals from primary care to community-based provision. The experiences shared by TSO staff indicated that effective social prescribing relies on fostering and sustaining a network of relationships between services, where ongoing collaboration and mutual trust are essential.

This study challenges commissioning bodies and policymakers to think differently about how social prescribing is structured and resourced. Rather than focusing predominantly on the quantity of referrals made, or the speed with which individuals are moved between services, attention needs to be paid to the quality of the relationships that underpin this work. This includes addressing long-standing structural imbalances between statutory and third sector services, ensuring that TSOs are not only adequately funded but also included in strategic planning and decision-making processes. In essence, what this study highlights is that social prescribing is not a process of referral and service use. Rather it is a community of practice, characterised by relational aspects, shared purpose, and collective ownership of challenges and solutions.

In our study, CLWs were, in the main, well regarded by TSOs. Referrals by them were mostly appropriate and timely. Successful referrals were those where CLWs practised intentional rather than scattergun placing of clients. Engagement in community services was more likely when CLWs accompanied clients to sessions and worked collaboratively with TSOs. Westlake et al. (38) also reported the importance of CLWs “holding” clients before their transition to the third sector. For better outcomes, clients had to be willing over time to take independent action to support their own health and wellbeing. Bhatti et al. (17) and Evers et al. (39) have also found that individual-level improvements in health outcomes could broadly be explained by self-determination theory.

Some TSOs said they could help to shape social prescribing arrangements from a “bottom-up” perspective, capitalising on their asset-based community development approach. They saw the CLW service as a stepping stone to a broader “citizen-based” health and welfare system, one that was not reliant on a “GP prescription.” Moving from a prescription mindset to a citizen model would involve TSOs and clients co-producing future social prescribing initiatives as well as influencing their adaptation and adjustment. However, as Lejac (6) reported, the capacity of community organisations to build these types of holistic systems is limited by several factors, including resource pressures, attitudes and historical ways of working.

More sustainable funding was the clarion call of many TSOs. With few revenue streams and facing large cuts, participants anticipated many community organisations would not survive or meet their goals in 2025. Resource limitations created instability for TSOs affecting service continuity and hindering long-term plans. There were also challenges in recruiting and retaining skilled staff with concomitant loss of knowledge and expertise. TSOs also noted that reductions in funding would impact the CLWs by providing them with fewer services to link to. Importantly, short-term funding undermined the sustained delivery of services, particularly vital for individuals requiring ongoing support to achieve the best outcomes.

Despite the third sector’s scale and reach in social prescribing, there was a perceived power imbalance between statutory and community services that undermined true partnership working. Cuts in funding emphasised the various challenges TSOs faced in relation to equity, including disparities in representation and access to opportunities. Others (22, 31) have also called for structural funding both at national and local level to support social prescribing, particularly for the third sector. Frequent closures and changes in available community activities not only undermine client trust and ability to engage in services but also make it more difficult for link workers to keep their knowledge up to date regarding available provision (40).

The evidence from this research suggests that fostering long-term partnerships would better meet community needs and lead to improvements in outcomes for social prescribing interventions. Relationships between the CLW service and TSOs were in many ways transactional in our study. Communication links were often reportedly one-way, with TSOs requesting more reciprocal interactions. In the cases where TSOs had forged collaborative exchanges with CLWs these were usually the result of long-lasting connections between individuals. Skivington et al. (32) reported similar findings in their study of 2018.

Different CLW models exist across the UK and internationally, but an innovative element found in this study was CLWs being employed directly by TSOs, albeit within a complex management structure. This arrangement helped to convey a community-centred approach with CLWs based in neighbourhood GP practices, responsive to client needs and knowledgeable about local provision. As we found, however, joint working at this level was not trouble-free. New interventions at the interface between health and social care can take many years to embed and require collaborative leadership, good team dynamics, and commitment to continuous review (41).

TSOs reported their services to be in extremely high demand, particularly for mental health needs. They worked hard to accommodate clients, helping them find purpose and build community networks. Fostering self-determination was perceived as a long-term endeavour. From TSO perspectives, collaborating more fully with the CLW service would optimise client outcomes although they recognised this may involve CLWs working outside their currently defined role.

### Implications

Realist evaluations of TSO involvement in CLW services may help to demonstrate evidence for what works, for whom and why but building an evidence base also requires agreement over appropriate measures of success. This study appeared to show a discrepancy between a “health-led” and a “community-led” approach to understanding and reporting outcomes. As Husk et al. (29) reminds us, not only do criteria need to be realistic and useful but effectiveness will also be dependent on complex interactions between clients, contexts, resources and services.

Focusing purely on process data (such as referral rates and linkages) fails to capture changes in clients’ wellbeing and social circumstances, as well as longer-term benefits such as reduced health care usage or return to paid employment. Use of a theory of change or logic model explicitly incorporating such outcomes could ensure that stakeholder views as well as systemic factors are incorporated into quality improvement initiatives of the CLW service going forward (42).

Working together requires commitment to a shared purpose with built-in opportunities and structures to support TSO and CLW staff collaboration. Future developments should focus on the CLW model as a long-term programme with TSOs involved in strategic planning and service development. The CLW service also needs to work more closely with TSOs as well as the individuals who use communities to fully understand their experiences and priorities. Implementing shadowing opportunities within TSOs for CLWs, and reciprocal arrangements for TSO staff, may help improve operational understanding, build relationships and strengthen referral quality.

Developing the CLW service as a community of practice could also help stakeholders come together, build relationships, share ideas, and support one another. Trust and ownership take time to grow, but facilitated well, could deliver shared learning, joint working on specific projects, and exchanges of best practice.

Social prescribing could play a much greater role in the public health landscape, extending its reach and prominence. The evidence from this research suggests that the third sector can grow locally resilient and self-supporting communities that encourage self-determination and wellbeing. We need to ensure we create more enabling environments to facilitate social prescribing practice, focusing on what works, and exploring practical ways of supporting positive collaboration.

### Strengths and limitations

A key strength of this work was its focus on the perspectives of TSOs in evaluating CLW social prescribing models. This is an under-researched area of study. Realist methods help to make explicit the contextual factors affecting an intervention and the mechanisms that drive specific outcomes. This type of approach aids understanding of the successful and less successful elements of programmes to improve future delivery. Interviewing link workers and clients would have provided a counterbalance to the third sector experience privileged in this study, although other studies have investigated these topics (38, 43). We might also have found different results had we undertaken the research in another region. However, the demographic and cultural similarities of Scotland to other nations undertaking CLW and social prescribing work means these findings have wider applicability. The short-term funding model is a major barrier for the third sector although announced cuts in revenue during the research may have generated more negative criticism than expected about the CLW service.

## Conclusion

TSOs are uniquely placed to support the health and wellbeing of clients, particularly in socio-economically deprived communities. Social prescribing is not a quick fix to tackle the challenges faced by primary care services. For social prescribing to be successful it will require sustained change, supported by long term investment. CLWs collaborating with TSOs have considerable potential to embed innovative citizen-centred support into Scotland’s public services, bridging the gap between clinical and non-medical support. Coproduction, including all partners and community users, in the design and implementation of social prescribing models going forward, is a key mechanism to strengthen partnership working between the CLW service and the third sector.

## Data Availability

The raw data supporting the conclusions of this article will be made available by the authors, without undue reservation.
